# Effect of resuscitative endovascular balloon occlusion of the aorta in nontraumatic out-of-hospital cardiac arrest: a multinational, multicenter, randomized, controlled trial

**DOI:** 10.1186/s13063-024-07928-x

**Published:** 2024-02-13

**Authors:** Hee Eun Kim, Sheng-En Chu, You Hwan Jo, Wen-Chu Chiang, Dong-Hyun Jang, Chin-Hao Chang, So Hee Oh, Hsuan-An Chen, Seung Min Park, Jen-Tang Sun, Dong Keon Lee

**Affiliations:** 1 Department of Emergency Medicine, Seoul National University Bundang Hospital, 13620, 82, Gumi-ro 173beon-gil, Bundang-gu, Seongnam-si, Gyeonggi-do, Republic of Korea; 2 Department of Emergency Medicine, Seoul National University College of Medicine, Seoul, Republic of Korea; 3 Department of Emergency Medicine, Far Eastern Memorial Hospital, No. 21, Sec. 2, Nan-Ya South Rd, Ban-Qiao Dist., New Taipei City, Taiwan; 4 Department of Public Healthcare Service, Seoul National University Bundang Hospital, Seongnam, Republic of Korea; 5 Department of Emergency Medicine, National Taiwan University Hospital Yun-Lin Branch, Yunlin, Taiwan; 6 National Taiwan University Hospital Statistical Consulting Unit, Taipei, Taiwan; 7 Medical Research Collaborating Center, SMG-SNU Boramae Medical Center Seoul, Seoul, Republic of Korea

**Keywords:** Cardiac arrest, Cardiopulmonary resuscitation, Resuscitative endovascular balloon occlusion of the aorta, REBOA, Out-of-hospital cardiac arrest

## Abstract

**Background:**

Out-of-hospital cardiac arrest (OHCA) is a significant public health issue worldwide and is associated with low survival rates and poor neurological outcomes. The generation of optimal coronary perfusion pressure (CPP) via high-quality chest compressions is a key factor in enhancing survival rates. However, it is often challenging to provide adequate CPP in real-world cardiopulmonary resuscitation (CPR) scenarios. Based on animal studies and human trials on improving CPP in patients with nontraumatic OHCA, resuscitative endovascular balloon occlusion of the aorta (REBOA) is a promising technique in these cases. This study aims to investigate the benefits of REBOA adjunct to CPR compared with conventional CPR for the clinical management of nontraumatic OHCA.

**Methods:**

This is a parallel-group, randomized, controlled, multinational trial that will be conducted at two urban academic tertiary hospitals in Korea and Taiwan. Patients aged 20–80 years presenting with witnessed OHCA will be enrolled in this study. Eligible participants must fulfill the inclusion criteria, and written informed consent should be collected from their legal representatives. Patients will be randomly assigned to the intervention (REBOA-CPR) or control (conventional CPR) group. The intervention group will receive REBOA and standard advanced cardiovascular life support (ACLS). Meanwhile, the control group will receive ACLS based on the 2020 American Heart Association guidelines. The primary outcome is the return of spontaneous circulation (ROSC). The secondary outcomes include sustained ROSC, survival to admission, survival to discharge, neurological outcome, and hemodynamic changes.

**Discussion:**

Our upcoming trial can provide essential evidence regarding the efficacy of REBOA, a mechanical method for enhancing CPP, in OHCA resuscitation. Our study aims to determine whether REBOA can improve treatment strategies for patients with nontraumatic OHCA based on clinical outcomes, thereby potentially providing valuable insights and guiding further advancements in this critical public health area.

**Trial registration:**

ClinicalTrials.gov NCT06031623. Registered on September 9, 2023

**Supplementary Information:**

The online version contains supplementary material available at 10.1186/s13063-024-07928-x.

## Introduction

### Background and rationale {6a}

Out-of-hospital cardiac arrest (OHCA) is a medical emergency that remains a public health issue worldwide [1]. The mainstay treatment for cardiac arrest involves immediate attention and treatment, including early recognition, high-quality chest compression, early defibrillation for shockable rhythm, early epinephrine administration, and immediate advanced airway access [2, 3]. With the development of guidelines for the treatment of cardiac arrest, numerous efforts have been made worldwide to increase survival rates and improve prognosis in patients with OHCA. However, despite such measures, the survival rates of patients with OHCA remain < 10% in several countries, and the proportion of patients with good neurological prognosis at discharge is even lower [1].

Cardiopulmonary resuscitation (CPR) for OHCA aims to reduce ischemic damage during cardiac arrest via continuous blood flow to the vessels supplying the vital organs. In particular, the maintenance of coronary blood flow is directly associated with the possibility of achieving the return of spontaneous circulation (ROSC), making it a crucial goal of CPR. However, even with high-quality chest compression, the rate of coronary blood flow usually remains < 30% of that before cardiac arrest [4, 5].

Several studies have been conducted on methods that can improve coronary blood flow during CPR. Resuscitative endovascular balloon occlusion of the aorta (REBOA) is a procedure that is traditionally used for temporary hemorrhage control in patients with trauma by occluding the aorta via balloon dilation [6]. In cases of nontraumatic cardiac arrest, REBOA adjunct to CPR can increase coronary perfusion pressure (CPP) by occluding the aorta and rerouting blood circulation to the heart and brain rather than other organs, thereby ultimately help achieving ROSC [7]. By applying REBOA in cases of nontraumatic cardiac arrest, several animal studies have shown positive results for coronary blood flow in terms of hemodynamics [8]. Further, recent trials have revealed that the application of REBOA in humans has promising outcomes [9, 10]. However, despite such promising results in terms of hemodynamic changes, research on the effect of REBOA on clinical outcomes in patients with nontraumatic OHCA is lacking.

### Objectives {7}

The current study aims to compare the clinical outcomes of CPR with REBOA and conventional CPR in patients with nontraumatic adult OHCA in the hospital stage.

### Trial design {8}

**Fig. 1:**
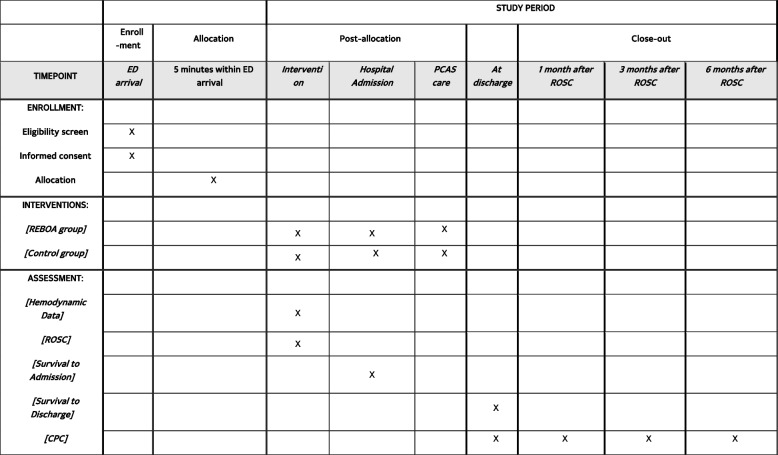
Schedule of enrollment, interventions, and assessments according to the Standard Protocol Items: Recommendations for Interventional Trials (SPIRIT) guideline

**Fig. 2:**
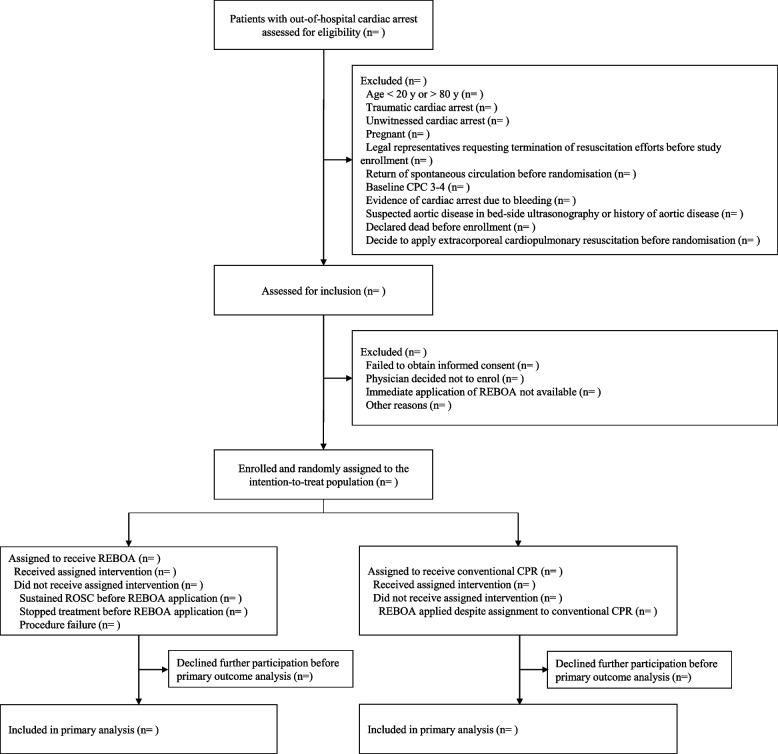
Study flow

The REBOA trial was designed as a parallel, randomized, controlled, multinational trial and registered at ClinicalTrials.gov (National Clinical Trial number: NCT06031623). Our study design is based on the SPIRIT 2013 Checklist (Additional file 1) [11]. Figure 1 shows the enrollment schedule, interventions, and study duration. Figure 2 depicts the study algorithm.

## Methods: participants, interventions, and outcomes

### Study setting {9}

This study will be conducted at two urban academic tertiary hospitals in Korea (Seoul National University Bundang Hospital (SNUBH)) and Taiwan (Far Eastern Memorial Hospital (FEMH)). SNUBH, a tertiary medical center in Seongnam in the southern Gyeonggi area, has a population of 3.1 million residents. As a 1337-bed capacity hospital, SNUBH records 70,000 emergency department (ED) visits annually. FEMH, the only tertiary medical center in New Taipei City, Taiwan, serves over 1.6 million residents in the western area of Greater Taipei. As a 1415-capacity hospital, FEMH records approximately 10,000 ED visits monthly, which includes approximately 25 cases of nontraumatic OHCA.

The emergency medical system (EMS) in South Korea is government-based and operates on a multi-tiered dispatch system for suspected cardiac arrests. Two teams, each comprising two to three members and located closest to the scene, are dispatched. The first team, upon arrival, initiates basic life support, which includes chest compressions (with a depth of 5 cm at a compression rate of 100–120 per minute), bag-mask ventilations, and the use of an automated external defibrillator (AED). Upon the second team’s arrival, advanced life support (ALS) is initiated if feasible, involving gaining intravenous access, administering epinephrine, and securing an advanced airway (through either a supraglottic airway or endotracheal intubation). ALS, if provided, is guided by an emergency physician via a video call. If it is determined during CPR at the scene that ROSC is unlikely to be rapidly achieved, preparations for transport immediately commence. Concurrently, upon the decision to initiate transport, notification is sent to the ED of the receiving hospital. This includes the patient’s medical history and the details of the arrest circumstances identified thus far. All patients are transported to the hospital for further resuscitation.

In the prehospital setting of Taiwan, when an emergency call is received, the dispatcher initially sends the first ambulance to the scene, equipped with a minimum of two emergency medical technicians (EMTs). If the dispatcher confirms the event as an OHCA during the call, additional ambulances, potentially a second or even a third, are dispatched to the site. Simultaneously, the hospital is notified to commence preparation. Following the protocol of the New Taipei City Fire Department, these EMTs are required to deliver advanced life support to all patients with OHCA before reaching the ED. This protocol includes mechanical chest compression (using LUCAS® 2, Stryker Medical, Kalamazoo, MI) at the center of the chest, maintaining a rate of 100 compressions per minute and a depth of 5 cm. Advanced airway management, involving either endotracheal intubation or a supraglottic airway; defibrillation; and resuscitative medication administration (e.g., epinephrine or amiodarone) through intravenous or intraosseous routes if available are part of the protocol. Following the initial CPR cycle, unless a prior “do not resuscitate” order is in place, all patients are transported to the hospital for further resuscitation.

### Eligibility criteria {10}

Written informed consent should be obtained from the legal representative prior to the research. All patients with witnessed nontraumatic adult OHCA who arrive at the ED from 9 am to 5 pm on working weekdays in each country’s time zone will be assessed for eligibility.

### Inclusion criterion

The inclusion criterion is patients aged 20–80 years with nontraumatic witnessed OHCA, arriving at the ED between 9 am and 5 pm (in each country).

### Exclusion criteria

The exclusion criteria include the following: (1) patients aged below 20 years old or over 80 years old; (2) those with traumatic cardiac arrest; (3) those with unwitnessed cardiac arrest; (4) pregnant patients; (5) those who have already achieved ROSC upon arrival at the ED; (6) those with a precardiac arrest cerebral performance category (CPC) score of 3–4; (7) those showing evidence of cardiac arrest caused by bleeding (e.g., gastrointestinal bleeding); (8) those suspected of aortic diseases, such as dissection, intramural hematoma, or aneurysm, using bedside ultrasound performed immediately after ED arrival or have a previous history of aortic disease; (9) those whose legal representatives request termination of resuscitation efforts before study enrollment; (10) those declared dead at scene before enrollment; and (11) those who meet the criteria for extracorporeal CPR (ECPR) and have been decided to receive ECPR. ECPR is applicable when all of the following criteria are met: precardiac arrest CPC score of 1–2, witnessed cardiac arrest with bystander CPR, aged 20–70 years, initial shockable rhythm, ECMO pump-on available within 60 min of cardiac arrest onset, and absence of end-stage diseases, such as cancer, liver cirrhosis, or end-stage renal failure.

Patients will be evaluated for eligibility based on patient history for most items in the exclusion and inclusion criteria. Due to the EMS systems in Korea and Taiwan notifying the ED of incoming cardiac arrests 5–15 min prior to arrival, along with the patient’s history and circumstances of the cardiac arrest at the scene, clinicians will be able to pre-assess whether the patient meets the eligibility criteria to a certain extent.

### Need for informed consent {26a}

As per the Declaration of Helsinki, obtaining informed consent is mandatory for any study involving human participants [12]. However, in the context of research on cardiac arrest research, securing informed consent from unconscious patients presents a considerable challenge [13]. Recognizing the necessity for prompt resuscitation, the United States Food and Drug Administration has outlined provisions to exempt the need for informed consent in certain emergency medical research scenarios [14].

At SNUBH, informed consent will be obtained upon ED arrival. Researchers will explain the purpose of the study, the risks and benefits of the intervention, and the possible complications. As patients with OHCA are unconscious during enrollment, written informed consent will be obtained from legal representatives; this consent form will include details about the study and provide information on how to reach the study investigator or ethics review board for any questions or concerns related to the research. If the patient regains consciousness and is adequately alert to comprehend the process, the researcher will explain the study protocol to the patient, and informed consent will be obtained.

The current study was approved by the Institutional Review Board (IRB) of FEMH. In accordance with the abovementioned provisions, the IRB of FEMH waived the need for informed consent, irrespective of whether the trial participants were assigned to the intervention (REBOA-CPR) or control (conventional ACLS) group. However, if a participant achieves sustained ROSC, the researchers will then be required to obtain written informed consent from the participant’s legally authorized representative.

### Need for additional consent for the collection and use of participant data and biological specimens {26b}

Biological specimens will not be collected. All data collected will be deidentified and then stored in an online database, which will only be available to the investigators with pre-authorized ID and password.

### Interventions

#### Explanation regarding the choice of comparators {6b}

This trial aims to examine the clinical impact of REBOA in patients with nontraumatic adult OHCA. Accordingly, patients will be randomly assigned to the intervention or control group with the intention to treat. The intervention group will receive REBOA in addition to conventional ACLS. Conventional ACLS will be provided according to the 2020 American Heart Association (AHA) guidelines [3]. In both study institutions, invasive hemodynamic monitoring using arterial catheterization is a routine practice during ACLS. The control (conventional CPR) group will receive conventional ACLS according to the 2020 AHA guidelines with invasive hemodynamic monitoring.

#### Intervention description {11a}

In SNUBH, ACLS will be initiated, and informed consent will be obtained from legal representatives simultaneously for eligible patients with OHCA upon ED arrival. At FEMH, eligible patients with OHCA will be enrolled in the study starting ACLS as soon as they arrive at the ED, as the IRB waived the acquisition of informed consent. After enrollment, patients will be randomly assigned to the intervention or control group.

#### REBOA insertion

While performing ACLS, the common femoral artery is accessed using an 8-F sheath catheter (Radifocus Introducer II, Terumo, Tokyo, Japan) based on the Seldinger technique under ultrasound guidance. Arterial catheterization is a routine intervention during ACLS at both institutions. Hence, an arterial catheter will be inserted, and arterial blood pressure (ABP) levels will be continuously monitored invasively during ACLS in both groups.

In the REBOA-CPR group, the REBOA catheter (Rescue Balloon, Tokai Medical Products, Aichi, Japan) will be inserted through the sheath catheter. To position the REBOA catheter at zone 1, the length from the site of insertion to the site immediately distal to the xiphoid process will be measured, and the REBOA catheter will be placed at the measured length along the presumed pathway of the artery. If possible, an abdominal ultrasonography or portable chest radiography machine will be used to confirm whether the REBOA catheter is placed adequately. After placing the REBOA catheter, the balloon will be cautiously inflated until the balloon diameter reaches 25 mm or until the physician experiences resistance. ACLS will be continuously provided with the balloon in an inflated state. After REBOA catheter inflation, ABP levels will be monitored at the tip of the REBOA catheter.

If the femoral artery is accessed via the sheath catheter and the patient is included in the intervention group, the REBOA catheter will be inserted immediately.

#### Hemodynamic monitoring protocol

ABP levels, central venous pressure (CVP) levels, electrocardiogram, and end-tidal carbon dioxide (ETCO_2_) levels will be continuously monitored and recorded during ACLS. ABP levels will be continuously monitored using the sheath catheter and REBOA catheter tip before and after balloon inflation, respectively. CVP levels will be measured via a central line placed during ACLS in the right internal jugular vein or subclavian vein under ultrasound guidance. ETCO_2_ levels will be monitored during ACLS to monitor the quality of chest compression and estimate cardiac output as an adjunct to ABP levels [15].

#### Post-resuscitation care

If the patient achieves ROSC, the REBOA balloon will be gradually deflated based on the discretion of the attending physician, if they judge that the risk of rearrest in a few minutes is low. Patients in both groups who achieved ROSC will receive postcardiac arrest care, including targeted temperature management (TTM), according to the 2020 AHA guideline [3]. If hypotension occurs, intravenous fluid and vasopressors will be administered as necessary.

#### Criteria for discontinuing or modifying the allocated interventions {11b}

Any participant or legal representatives who want to withdraw from the study will be allowed to do so without any consequences. If the legal representative of the patient chooses to withdraw from the study, the patient will continue to receive conventional CPR, similar to that provided to patients in the control group.

#### Strategies to improve intervention adherence {11c}

To improve adherence, regular meetings will be conducted to review and ensure protocol adherence. Regarding any protocol violations that are identified after intervention, additional meetings will be conducted to review each case.

#### Relevant concomitant care permitted or prohibited during the trial {11d}

There are no restrictions regarding concomitant care during the trial. Implementing the intervention will not require alteration to the usual management of cardiac arrest, which follows the 2020 AHA guidelines and applies to both trial arms.

#### Provisions for post-trial care {30}

The provisions for post-trial care in this study are within the scope of postcardiac arrest care according to the 2020 AHA guidelines. In addition to TTM, care will be based on the presumed etiology of cardiac arrest for each patient. In patients who achieved ROSC, any subsequent adverse events related to REBOA catheter insertion, such as vessel injury, lower limb ischemia, and hematoma/infection, which occurs at the insertion site of the REBOA catheter, will be monitored. In addition, possible related complications such as acute kidney which requires renal replacement therapy, acute respiratory distress syndrome, bacteremia, pneumonia, sepsis/septic shock, and paraplegia will be monitored.

### Outcomes {12}

#### Primary outcome

The primary outcome will be ROSC in the ED, which is represented by the number of patients who achieved ROSC regardless of sustained time.

#### Secondary outcomes

The secondary outcomes will be sustained ROSC (defined as ROSC maintained for at least 20 min); survival to admission; survival to discharge; neurological outcome at discharge as well as 1, 3, and 6 months after ROSC; and hemodynamic changes at 1, 2, 4, and 10 min after REBOA inflation. Data regarding complications associated with REBOA catheter insertion will also be considered as secondary outcomes. Information on neurological outcomes will be obtained using the CPC scale and Modified Rankin Scale (mRS). If the surviving patients are discharged, the research nurse will conduct phone interviews with them or their legal representatives to determine CPC and mRS scores at each time point. REBOA catheter insertion-related complications include direct or indirect adverse events such as malposition, insertion failure, hematoma/infection, femoral artery injury, lower limb ischemia, and aortic injury.

### Participant timeline {13}

**Fig. 3:**
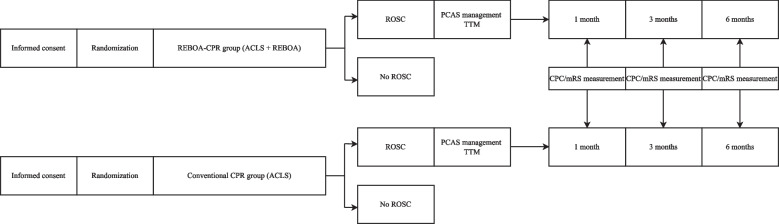
Participant timeline. ROSC, return of spontaneous circulation; PCAS, postcardiac arrest syndrome; TTM, targeted temperature management; CPC, cerebral performance category; mRS, Modified Rankin Scale

Figure 3 shows the participant timeline.

### Sample size {14}

The primary outcome will be ROSC. Current data supporting the sample size of this study are limited. According to a pilot study on the application of REBOA in patients with nontraumatic OHCA, which was performed at one of the two institutions involved in the current study, 40% of patients achieved ROSC in the ED [10]. According to a study on conventional CPR for patients with OHCA, which was conducted at the same institution, 21.4% of patients achieved ROSC in the ED [13]. We used these differences to estimate the sample size with alpha values of 0.05 and a power of 0.8 and revealed that 212 patients (106 in each group) will be required. Finally, with a dropout rate of 10%, 234 patients should be enrolled, with equal proportions in each arm.

### Recruitment {15}

Each hospital will assess all patients with nontraumatic adult OHCA for eligibility and continue to enroll patients until the specific sample size is reached. At least 50 patients will be enrolled competitively at each institution.

### Assignment of interventions: allocation

#### Sequence generation {16a}

The randomization sequence will be generated using the R statistical software (version 4.1.3; R Core Team, R Foundation for Statistical Computing, Vienna, Austria). The randomized permuted block design with block sizes of 2, 4, and 6 will be employed. Randomization will be stratified by institution, ensuring that an equal number of participants are assigned to each treatment group in each institution. The sequence will be implemented using the electronic case report form (CRF) software (MyECRF, LUNAAIR, Republic of Korea).

#### Concealment mechanism {16b}

Owing to the nature of the intervention, it will be challenging to blind the participating physicians during the study. Hence, no concealment mechanism will be employed. Randomization sequences will be concealed from the participating physicians. Group assignments will be blinded to the data analysts. Deidentified CRFs will be used to blind data analysts. Data from each group will be stored in electronic CRF as groups A and B. Thus, the data analyst will be blinded to the actual group.

#### Implementation {16c}

An independent statistician will generate an allocation sequence. The generated randomization sequence will be stored in electronic CRF (MyECRF, LUNAAIR, South Korea) and will not be released to the research team. If the emergency medical service team notifies the ED of the study institutions regarding incoming OHCA cases, the participating physician on call will assess the eligibility and enroll participants. After enrollment, the researcher will disclose the allocation information stored in electronic CRF to the participating physician.

### Assignment of interventions: blinding

#### Who will be blinded {17a}

Owing to the nature of the study, neither the researcher nor the patient or legal representatives will be blinded.

#### Procedure for unblinding if needed {17b}

The design is an open-label study, so unblinding will not occur.

### Data collection and management

#### Plans for the assessment and collection of outcomes {18a}

Demographic characteristics (such as age, sex, underlying medical condition, and CPC score before cardiac arrest), cardiac arrest details (time and place of arrest, witness of the arrest, bystander CPR and AED use, initial rhythm, EMS call time and arrival time, scene departure time, initial cardiac rhythm, established airway, chest compression via EMS, defibrillation energy and attempts, intravenous access, and medication administered), in-hospital information (time of ED arrival, time of central/arterial line insertion, established airway in the ED, medication administration, and ABP levels during ACLS), data regarding REBOA catheter insertion (insertion time and site, number of attempts, balloon inflation volume, and device-related complications), patient outcomes (ROSC, sustained ROSC, time and location of ROSC, survival to admission, survival to discharge, in-hospital mortality, length of hospital/intensive care unit stay, good neurological outcome at hospital discharge as well as 1, 3, and 6 months after ROSC), and adverse events will be collected. Tables 1 and 2 depicts the demographic characteresitics and outcomes that will be collected.

**Table 1:** Demographic characteristics of patients by group

	Total (*n* = )	REBOA group (*n* = )	Conventional group (*n* = )
Age			
Sex			
Comorbidity			
HTN			
DM			
CAOD			
HF			
Stroke			
Public place			
Bystander CPR			
Initial rhythm shockable			
Cardiac arrest etiology			
Prehospital no flow time (min)			
Prehospital low flow time (min)			
ED arrival to arterial line insertion interval (min)			
ED arrival to central line insertion interval (min)			

*HTN *Hypertension, *DM *Diabetes mellitus, *CAOD *Coronary artery obstructive disease, *HF *Heart failure, *CPR *Cardiopulmonary resuscitation, *ED *Emergency department

**Table 2:** Comparison of patient outcomes by group

	Total (*n* = )	REBOA group (*n* = )	Conventional group (*n* = )
Any ROSC			
Time to the 1st ROSC (min)			
Sustained ROSC			
Time to the 1st sustained ROSC			
The length of time a sustained ROSC has been maintained			
TTM			
CRRT			
CAG			
Length of hospital stay (days)			
Survival to admission			
Good CPC at			
Hospital discharge			
1 month after ED arrival			
3 months after ED arrival			
6 months after ED arrival			

*ROSC *Return of spontaneous circulation, *TTM *Targeted temperature management, *CRRT *Continuous renal replacement treatment, *CAG *Coronary artery angiography, *CPC *Cerebral performance category, *ED *Emergency department

The definition of cardiac arrest data will be recorded based on the Utstein Resuscitation Registry Templates for OHCA [16]. The primary outcome will be ROSC at any point during the resuscitation attempt in the ED. Sustained ROSC will be defined as maintaining the patient’s spontaneous circulation without chest compressions for > 20 min. Further, good neurological outcomes are defined as CPC 1 and 2 [17]. Changes in hemodynamic parameters after REBOA insertion will be analyzed over time by obtaining a continuous blood pressure signal.

#### Plans to promote participant retention and complete follow-up {18b}

Considering that our primary outcome is any ROSC in the ED, no loss to follow-up until the primary outcome is checked after enrollment is expected. To assess secondary outcomes, the following plans will be implemented to promote participant retention and complete follow-up.

The researcher in charge of each institution will follow up with the patients who achieved ROSC and were discharged from the hospital to collect CPC scores at 1, 3, and 6 months. To reduce loss to follow-up as much as possible, the study institution will make every reasonable effort to follow up with patients for the entire study period, including the discussion of their health status when assessing CPC via phone.

Any decisions to withdraw from the study made by the trial participant or their legally authorized representative will be meticulously recorded, along with the reasons and precise timing of such actions. If a participant becomes inaccessible during the follow-up period, the corresponding data will be marked as missing in the analysis. Ultimately, these exhaustive details regarding withdrawal will be incorporated into the Consolidated Standards of Reporting Trials flow diagram, thus providing a transparent and integrated overview of participant retention throughout the study.

#### Data management {19}

Data will be entered into an electronic CRF, which will be accessible only to the researcher in charge. Each data item will have a designated range (e.g., 18–150 years for age). If the value for a particular item falls outside this range, an immediate warning will be issued. Furthermore, if the value lies within the appropriate range but does not meet specific conditions (e.g., the recorded time of arterial line insertion precedes the arrival time at the ED), a query will be forwarded to the researcher in charge for necessary corrections.

#### Confidentiality {27}

Data will be stored based on the study number assigned to each participant. Paper CRFs will be stored in locked file cabinets in the laboratory. Data will also be transferred to electronic CRFs by the researcher in charge, and only researchers who have been authorized in advance will have access to this system.

#### Plans for collection, laboratory evaluation, and storage of biological specimens for genetic or molecular analysis in this trial/future use {33}

This study will not require biological specimens for genetic or molecular analysis.

## Statistical methods

### Statistical analysis of the primary and secondary outcomes {20a}

Data analysis will be performed using the R statistical software (version 4.1.3; R Core Team, R Foundation for Statistical Computing, Vienna, Austria). The Shapiro–Wilk test will be used to assess data normality. Continuous data that follow a normal distribution will be presented as the mean ± standard deviation and further analyzed using an independent *t*-test. In contrast, data not adhering to a normal distribution will be expressed as median and interquartile range and further evaluated using the Mann–Whitney *U* test. Categorical variables, including ROSC and survival, will be presented as frequencies (percentages) and analyzed using the chi-square test or Fisher’s exact test, as appropriate. For variables demonstrating statistically significant differences between the two groups, the disparity will be reported as either absolute difference or risk difference, depending on the variable type. The analysis of the primary outcome difference between the two groups will use both absolute difference and risk difference. Additionally, when applicable, the risk ratio will be presented as an additional measure, as appropriate. *P*-values of < 0.05 will be considered to indicate statistical significance. In an interim analysis, the significance level according to the O’Brien–Fleming approach will be used. A statistical analysis plan will be attached as a supplementary file (Additional file 2).

### Interim analysis {21b}

After the enrollment of the first 116 patients, an interim analysis will be performed according to the O’Brien–Fleming rule. A blinded interim analysis will be performed by an independent statistician. Both primary and secondary outcomes will be assessed using interim analysis. If there is a statistically significant difference in ROSC, sustained ROSC, or survival to discharge between the two groups, with a significance level of 0.005, the trial will be considered for termination.

The sample size will be recalculated based on the assumption that the current difference in the primary endpoint between the two groups will persist. (1) If the sample size required to prove a difference exceeds three times the initially planned sample size (with a power of 0.80 and a significance level of 0.05), the Trial Steering Committee (TSC) will decide whether to continue the trial. (2) If the recalculated sample size falls within the range of 348–696 (150–300% of the original sample size), the TSC will discuss whether the study should proceed with the new sample size. (3) If the revised sample size is < 348 (150% of the original sample size), the study will proceed with the adjusted sample size.

### Methods for additional analysis {20b}

Additional subgroup analysis will aim to investigate factors potentially leading to disparities in treatment outcomes, thereby indicating that these effects may vary based on the distinct clinical characteristics of patients throughout the study. This detailed evaluation will be conducted in various potential subgroups of age, sex, administration of CPR using a bystander, initial rhythm (categorized as shockable or nonshockable), location of the arrest (public vs. nonpublic), presumed cause of arrest, time elapsed from arrest to arrival at the ED, and the institution where the patient received treatment (SNUBH or FEMH).

### Analysis method for handling protocol nonadherence and statistical methods for handling missing data {20c}

For statistical analysis, two sets will be utilized: intention-to-treat set and per-protocol set. The intention-to-treat set will analyze patients based on the group they were assigned to after randomization, regardless of the actual randomized treatment received. The per-protocol set will identify patients who received or did not receive REBOA (REBOA or conventional group, respectively) for analysis.

Authors expect no missing data for the primary outcome (any ROSC), which is immediately determined in all patients after the termination of ACLS. When reporting patient clinical characteristics and secondary outcomes, the primary approach to handling missing data will be the direct deletion method. However, in cases where a significant amount of missing data exists for specific clinical characteristics, multiple imputation will be considered as an alternative approach. If multiple imputation is employed for any variable, it will be explicitly stated in the results.

### Plans to provide access to the full protocol, participant-*level data, and statistical code {31c}*

The patient-level dataset will not be accessed by the public.

### Oversight and monitoring

#### Composition of the coordinating center and TSC {5d}

Four committees will play roles in the research: Trial Steering Committee, Trial Management Group, Data Monitoring Committee, and the Data Coordinating Center.

##### Trial Steering Committee (TSC)

The TSC, comprising six members, includes an independent chair, an independent statistician, an independent member, and three nonindependent members from each participating institution. The TSC oversees patient safety, ensures data integrity, and makes executive decisions, such as modifications to or discontinuation of the study protocol.

##### Trial Management Group (TMG)

The TMG, consisting of two clinicians from each participating institution, is responsible for overseeing the day-to-day execution of trial performance. Communication within the TMG occurs online as necessary.

##### Data Monitoring Committee (DMC)

The DMC, comprising four members, including one statistician, all independent from the trial, reviews efficacy and safety data, conducts interim analyses, oversees recruitment, assesses data quality, monitors protocol deviations, and addresses safety and adverse events. The DMC meets every 6 months and provides recommendations to the TSC.

##### Data Coordinating Center (DCC)

The DCC, with two members—one from each participating institution—regularly assesses trial data quality using electronic CRF (eCRF) software.

#### Composition, role, and reporting structure of the DMC {21a}

The trial will be independently monitored by the DMC, which will review the safety and efficacy of the trial and send biannual reports to the TSC.

#### Reporting of adverse events {22}

Adverse events will be monitored from randomization to hospital discharge. If any adverse events occur, patients will be treated immediately according to routine practice and followed up until resolution or treatment termination. Such adverse events will be reported by the DMC to the TSC and IRB.

All adverse events will be recorded in CRFs for each case. Table 3 presents a list of adverse events that will be recorded. All adverse events and complications associated with REBOA catheter insertion will be analyzed and reported as secondary outcomes.

**Table 3:** Resuscitative endovascular balloon occlusion of the aorta (REBOA) procedure data and adverse events

	Total (*n* = )	REBOA group (*n* = )
ED arrival to REBOA inflation interval (s)		
Insertion depth of REBOA catheter (cm)		
Amount of NS used for ballooning (cc)		
Adverse events		
Device-related complications		
Malposition		
Insertion failure		
Insertion site hematoma		
Insertion site infection		
Balloon rupture		
Catheter removal failure		
Vascular access-related complications		
Femoral artery injury		
Lower limb ischemia		
Amputation		
Aorta injury		
Adverse events		
AKI with dialysis		
ARDS		
Pneumonia		
Sepsis/septic shock		
Stroke/CVA		
Paraplegia		
Myocardial infarction		
Multiorgan failure		
Neurologic deficit secondary to spinal cord ischemia		
Ventilator days		
Length of ICU stay		
Length of hospital stay		
In hospital stay		
Mortality, hours after admission		
Mortality, hospital days		

*ED *Emergency department, *REBOA* Resuscitative endovascular balloon occlusion of aorta, *NS *Normal saline, *AKI* Acute kidney injury, *ARDS* Acute respiratory distress syndrome, *CVA* Cerebrovascular accident, *ICU* Intensive care unit

#### Frequency and plans for auditing trial conduct {23}

The DMC will convene every 6 months to analyze and review trial safety. Basic patient demographic characteristics, adequacy of patient recruitment, protocol deviations, adequacy of follow-up, and serious adverse events will be reported. Although the meeting will be conducted independently from the study investigators, any information or issues that are deemed necessary will be reported to the TSC and IRB.

The electronic CRF software employs formulas and conditions to assess data quality. If any input value does not satisfy a condition, an automatic query will be raised, and the DCC will evaluate the patient’s original CRF (paper copy) and correct the input value if necessary. If an error occurs despite similar values in paper and electronic CRFs, then the TMG will be informed, and the error will be corrected and reported to the TSC. The DCC will also assess data completeness on a daily basis and may ask the TSC to add/modify/delete formulas and conditions if necessary.

#### Plans for communicating important protocol amendments to relevant parties {25}

Any modifications to the protocol that may affect the study conduct, potential patient benefits, or patient safety, including changes to the study objectives, study design, patient population, sample size, study procedures, or important administrative aspects, will require notification to the sponsor first. Subsequently, a copy of the revised protocol will be sent to the TSC. The revised protocol will undergo review by the TSC, and the final decisions made by the TSC will receive approval from IRB of each participating institution. Following approval, the revised protocol will be incorporated into the investigator site file, and the trial registration will be updated accordingly. Any deviations from the protocol will be thoroughly documented using a breach report form.

#### Dissemination plans {31a}

Our findings will be disseminated via journal publications and conference presentations.

## Discussion

To achieve ROSC in cardiac arrest, coronary perfusion should be restored [5, 18]. CPP, which is calculated as the difference between aortic and diastolic right atrial pressure levels during the relaxation phase of chest compressions, indicates myocardial perfusion and CPR quality [5, 18].

REBOA, a mechanical technique capable of increasing CPP, serves as an alternative to the currently used method [19]. Demonstrating promising outcomes in animal models and pilot studies, REBOA may offer a feasible alternative or supplementary treatment approach to achieve favorable clinical results in patients with nontraumatic OHCA [20]. A study to observe the utility of REBOA in nontraumatic cardiac arrest is currently underway in Norway [21]. The primary outcome of this study is the proportion of ROSC, with a goal of enrolling 200 patients with a 1:1 allocation. However, this study is focused on the application of REBOA in the prehospital setting. The upcoming parallel-group, randomized controlled trial will be conducted at two academic tertiary hospitals in South Korea and Taiwan, focusing on hospital settings. Further, it will evaluate whether REBOA can significantly improve the current OHCA treatment practices, focusing primarily on achieving ROSC and secondarily on evaluating sustained ROSC and neurological outcomes in the patient population.

In addition, there has been a recent report regarding the use of REBOA in trauma patients with exsanguinating hemorrhage [22]. The study suggests that in cases of trauma-related exsanguinating hemorrhage, REBOA did not reduce mortality. Since the primary focus of this study is on patients with traumatic hemorrhage, a disease entity distinct from nontraumatic cardiac arrest, interpreting results from this study to nontraumatic cardiac arrest patients may be challenging. Despite the differences in subjects, it is crucial not to overlook the possibility that similar results may emerge in our current study.

By investigating REBOA as a means to enhance CPP during CPR, our study aims to explore alternative treatment protocols that may contribute to more effective outcomes in patients with nontraumatic OHCA. The research results may provide valuable insights and add to the growing body of evidence that may help improve treatment strategies for nontraumatic OHCA. Ultimately, these study findings can be an important step toward further advancements and innovations in this important area of public health.

## Trial status

Recruitment will start in October 2023 and end in September 2025. The current version of the protocol is version 1.0 (September 8, 2023).

### Supplementary Information

**Additional file 1.** SPIRIT 2013 Checklist.

**Additional file 2.** Statistical analysis plan.

**Additional file 3.** Informed consent (SNUBH).

**Additional file 4.** Informed consent (FEMH).

## Data Availability

The study results will be published through conferences and scientific publications in medical journals related to emergency medicine or resuscitation. Deidentified data will be available for online provision upon reasonable request.

## Abbreviations

ACLS: Advanced cardiovascular life support
ALS: Advanced life support
AHA: American Heart Association
ABP: Arterial blood pressure
AED: Automated external defibrillator
CPR: Cardiopulmonary resuscitation
CRF: Case report form
CVP: Central venous pressure
CPC: Cerebral performance category
CPP: Coronary perfusion pressure
DCC: Data Coordinating Center
DMC: Data Monitoring Committee
ED: Emergency department
EMS: Emergency Medical System
ETCO_2_: End-tidal carbon dioxide
ECPR: Extracorporeal cardiopulmonary resuscitation
FEMH: Far Eastern Memorial Hospital
IRB: Institutional Review Board
mRS: Modified Rankin Scale
OHCA: Out-of-hospital cardiac arrest
REBOA: Resuscitative endovascular balloon occlusion of the aorta
ROSC: Return of spontaneous circulation
SNUBH: Seoul National University Bundang Hospital
TTM: Targeted temperature management
TMG: Trial Management Group
TSC: Trial Steering Committee

## References

[1] Kiguchi T, Okubo M, Nishiyama C, Maconochie I, Ong MEH, Kern KB (2020). Out-of-hospital cardiac arrest across the world: first report from the International Liaison Committee on Resuscitation (ILCOR). Resuscitation..

[2] Soar J, Böttiger BW, Carli P, Couper K, Deakin CD, Djärv T (2021). European Resuscitation Council Guidelines 2021: adult advanced life support. Resuscitation..

[3] Panchal AR, Bartos JA, Cabañas JG, Donnino MW, Drennan IR, Hirsch KG (2020). Part 3: Adult basic and advanced life support: 2020 American Heart Association Guidelines for Cardiopulmonary Resuscitation and Emergency Cardiovascular Care. Circulation..

[4] Bellamy RF, DeGuzman LR, Pedersen DC (1984). Coronary blood flow during cardiopulmonary resuscitation in swine. Circulation..

[5] Meaney PA, Bobrow BJ, Mancini ME, Christenson J, de Caen AR, Bhanji F (2013). Cardiopulmonary resuscitation quality: [corrected] improving cardiac resuscitation outcomes both inside and outside the hospital: a consensus statement from the American Heart Association. Circulation..

[6] Castellini G, Gianola S, Biffi A, Porcu G, Fabbri A, Ruggieri MP (2021). Resuscitative endovascular balloon occlusion of the aorta (REBOA) in patients with major trauma and uncontrolled haemorrhagic shock: a systematic review with meta-analysis. World J Emerg Surg..

[7] Nowadly CD, Johnson MA, Hoareau GL, Manning JE, Daley JI (2020). The use of resuscitative endovascular balloon occlusion of the aorta (REBOA) for non-traumatic cardiac arrest: a review. J Am Coll Emerg Physicians Open..

[8] Hutin A, Levy Y, Lidouren F, Kohlhauer M, Carli P, Ghaleh B (2021). Resuscitative endovascular balloon occlusion of the aorta vs epinephrine in the treatment of non-traumatic cardiac arrest in swine. Ann Intensive Care..

[9] Brede JR, Lafrenz T, Klepstad P, Skjærseth EA, Nordseth T, Søvik E (2019). Feasibility of pre-hospital resuscitative endovascular balloon occlusion of the aorta in non-traumatic out-of-hospital cardiac arrest. J Am Heart Assoc..

[10] Jang DH, Lee DK, Jo YH, Park SM, Oh YT, Im CW (2022). Resuscitative endovascular occlusion of the aorta (REBOA) as a mechanical method for increasing the coronary perfusion pressure in non-traumatic out-of-hospital cardiac arrest patients. Resuscitation..

[11] Chan A-W, Tetzlaff JM, Gøtzsche PC, Altman DG, Mann H, Berlin JA (2013). SPIRIT 2013 explanation and elaboration: guidance for protocols of clinical trials. BMJ..

[12] World Medical A (2013). World Medical Association Declaration of Helsinki: ethical principles for medical research involving human subjects. JAMA..

[13] Foëx BA (2001). The problem of informed consent in emergency medicine research. Emerg Med J..

[14] Biros MH, Fish SS, Lewis RJ (1999). Implementing the Food and Drug Administration’s final rule for waiver of informed consent in certain emergency research circumstances. Acad Emerg Med..

[15] Otlewski MP, Geddes LA, Pargett M, Babbs CF (2009). Methods for calculating coronary perfusion pressure during CPR. Cardiovasc Eng..

[16] Perkins GD, Jacobs IG, Nadkarni VM, Berg RA, Bhanji F, Biarent D (2015). Cardiac arrest and cardiopulmonary resuscitation outcome reports: update of the Utstein Resuscitation Registry Templates for Out-of-Hospital Cardiac Arrest: a statement for healthcare professionals from a task force of the International Liaison Committee on Resuscitation (American Heart Association, European Resuscitation Council, Australian and New Zealand Council on Resuscitation, Heart and Stroke Foundation of Canada, InterAmerican Heart Foundation, Resuscitation Council of Southern Africa, Resuscitation Council of Asia); and the American Heart Association Emergency Cardiovascular Care Committee and the Council on Cardiopulmonary, Critical Care. Perioperative and Resuscitation. Resuscitation..

[17] Jennett B, Bond M (1975). Assessment of outcome after severe brain damage. Lancet..

[18] Reynolds JC, Salcido DD, Menegazzi JJ (2010). Coronary perfusion pressure and return of spontaneous circulation after prolonged cardiac arrest. Prehosp Emerg Care..

[19] Mazzoli CA, Chiarini V, Coniglio C, Lupi C, Tartaglione M, Gamberini L, et al. Resuscitative endovascular balloon occlusion of the aorta (REBOA) in non-traumatic cardiac arrest: a narrative review of known and potential physiological effects. J Clin Med Res. 2022;11(3):4–6. 10.3390/jcm11030742. 10.3390/jcm11030742 PMC8836569 35160193

[20] Olsen MH, Olesen ND, Karlsson M, Holmlöv T, Søndergaard L, Boutelle M (2021). Randomized blinded trial of automated REBOA during CPR in a porcine model of cardiac arrest. Resuscitation..

[21] Brede JR, Skulberg AK, Rehn M, Thorsen K, Klepstad P, Tylleskär I, Farbu B, Dale J, Nordseth T, Wiseth R, Krüger AJ (2021). REBOARREST, resuscitative endovascular balloon occlusion of the aorta in non-traumatic out-of-hospital cardiac arrest: a study protocol for a randomised, parallel group, clinical multicentre trial. Trials..

[22] Jansen JO, Hudson J, Cochran C, MacLennan G, Lendrum R, Sadek S, Gillies K, Cotton S, Kennedy C, Boyers D, Ferry G (2023). Emergency department resuscitative endovascular balloon occlusion of the aorta in trauma patients with exsanguinating hemorrhage: the UK-REBOA randomized clinical trial. JAMA..

