# Supplementary material for: Effect of resuscitative endovascular balloon occlusion of the aorta in nontraumatic out-of-hospital cardiac arrest: a multinational, multicenter, randomized, controlled trial
Source: Trials. 2024 Feb 13;25:118. doi: 10.1186/s13063-024-07928-x (PMC10863125; doi:10.1186/s13063-024-07928-x)
Supplement: Supplementary file 2 — Additional file 2. Statistical analysis plan. [file 13063_2024_7928_MOESM2_ESM.docx]

Title: Effects of Resuscitative Endovascular Balloon Occlusion of the Aorta on Nontraumatic Out-of-hospital Cardiac Arrest (REBOA): A Multinational, Multicenter Randomized Controlled Trial

Trial Registration Number:

Current Version Number: Version 1.0

Release Date: September 17, 2023

Version History:

V1.00

**Contributors and Affiliations**

Seoul National University Bundang Hospital Dong Keon Lee

Seoul National University Bundang Hospital You Hwan Jo

Seoul National University Bundang Hospital Seung Min Park

Seoul National University Bundang Hospital Dong-Hyun Jang

Seoul National University Bundang Hospital Hee Eun Kim

Far Eastern Memorial Hospital Jen-Tang Sun

Far Eastern Memorial Hospital Sheng-En Chu

Far Eastern Memorial Hospital Xuan-An Chen

National Taiwan University Hospital Wen-Chu Chiang

National Taiwan University Hospital Statistical Consulting Unit Chin-Hao Chang

**Signatures**

# Introduction

## Background and Rationale

Out-of-hospital cardiac arrest (OHCA) is a medical emergency that remains a public health issue worldwide. The primary management approach for cardiac arrest emphasizes immediate attention and treatment, including early recognition, high-quality chest compressions, early defibrillation for shockable rhythms, early epinephrine administration, and advanced airway access as soon as possible. Apart from the development of these guidelines, substantial international efforts have been directed toward increasing survival rates and improving the prognosis of patients with OHCA. However, despite these undertakings, fewer than 10% of OHCA patients in many countries survive, and even fewer show good neurological prognosis at discharge.

Cardiopulmonary resuscitation (CPR) primarily aims to reduce ischemic damage during cardiac arrest by maintaining blood flow to vessels supplying vital organs. Maintaining coronary blood flow, which supplies blood to the heart, is directly associated with the likelihood of achieving return of spontaneous circulation (ROSC). However, even with high-quality chest compressions, coronary blood flow typically remains at less than 30% of the pre-cardiac arrest levels.

Several studies have sought to determine how to improve the coronary blood flow during CPR. Resuscitative endovascular balloon occlusion of the aorta (REBOA) is traditionally used in trauma patients for temporary hemorrhage control by occluding the aorta via balloon dilation. By occluding the aorta and redirecting blood circulation toward the heart and brain rather than other organs during cardiac arrests, REBOA can assist in increasing the coronary perfusion pressure, which consequently helps achieve ROSC. Several animal studies have shown that applying REBOA to nontraumatic cardiac arrest patients promotes positive hemodynamic results in terms of coronary blood flow, and recent trials on its application in humans have also shown promising results (Brede et al. 2019; Jang et al. 2022). However, despite these encouraging findings related to hemodynamic changes, research on the effects of REBOA on clinical outcomes in patients with nontraumatic OHCA remains limited.

## Study Objectives and Hypothesis

This trial primarily aims to evaluate the clinical impact of REBOA in nontraumatic OHCA patients and compare with that of conventional advanced cardiac life support (ACLS). The hypothesis of this study is that OHCA patients treated with REBOA are more likely to achieve ROSC than those receiving traditional ACLS alone.

# Study Methods

## Trail Design

This prospective, multicenter, open-label, parallel-group, randomized controlled trial will be conducted in two tertiary hospitals: Seoul National University Bundang Hospital in South Korea and Far Eastern Memorial Hospital in Taiwan. Patients who are transported intra-arrest and satisfy all inclusion criteria without any exclusionary factors will be promptly and randomly assigned on a 1:1 basis to either the REBOA CPR group or the conventional CPR group immediately upon arrival at the emergency department (ED). The primary aim of this study was to compare post-CPR prognosis between the two patient cohorts.

## Randomization

The randomization sequence will be generated using the R statistical software (version 4.2.3; R Core Team, R Foundation for Statistical Computing, Vienna, Austria). A randomized permuted block design with block sizes of 2, 4, and 6 will be used. Randomization will be stratified according to the institution, ensuring an equal number of participants assigned to each treatment group at each institution. The sequence will be implemented in electronic case report form software (MyECRF, LUNAAIR, Republic of Korea).

## Sample Size

- - 1. **Sample Size Calculation**

A pilot study on the application of REBOA in nontraumatic OHCA patients at one institution in the present study found that the rate of achieving ROSC in the ED was 40.0%. Another study involving the application of the conventional CPR method for OHCA patients at the same institution showed that the rate of achieving ROSC in the ED was 21.4%.

Using this difference, the sample size was calculated using an alpha of 0.05 and a beta of 0.8, which revealed that a total of 212 patients were required. Assuming a dropout rate of 10%, this study will seek to enroll 234 patients (117 per arm).

- - 1. **Number of Subjects per Center**

Patients will be enrolled competitively with a minimum requirement of 50 at each institution. This has been established to maintain statistical balance and analytical rigor, fostering a reliable and comparative examination of data from these distinct sites. This approach forms a key part of our statistical analysis plan and will aid in achieving meaningful and reliable results.

## Framework

The analytical framework of this study is designed to evaluate the comparability of outcomes between the two CPR strategies: CPR with REBOA and conventional CPR. Consequently, to thoroughly assess the comparability of outcomes, we undertake a two-tailed approach for our hypothesis testing.

## Statistical Interim Analyses and Stopping Guidance

After enrolling the first 116 patients, an interim analysis will be performed according to the O'Brien–Fleming rule. An independent statistician will perform a blinded interim analysis. The primary (ROSC) and secondary outcomes will be assessed in the interim analysis using a significance level of 0.005 following the O’Brien–Fleming approach.

The sample size will be recalculated based on the assumption that the current difference in the primary endpoint between the two groups will persist. (1) If the recalculated sample size needed to demonstrate a difference exceeds three times our initially planned sample size (assuming a power of 0.80 and significance level of 0.05), then the Trial Steering Committee (TSC) will decide whether to continue with the trial. (2) If this recalculated sample size ranges between 348 and 696, which is 150% to 300% of our initial estimate, then the TSC will discuss whether to adjust our study to reflect this new sample size. (3) If this revised calculation falls below 348, which is less than or equal to 150% of our original estimate, then we proceed with this adjusted number for our sample size.

## Timing of Final Analysis

The final analysis for this research study will occur 6 months after the enrollment of the last participant. This timeline was strategically devised to align with the sequential follow-ups planned at the end of the 1st, 3rd, and 6th months after enrollment. Meticulous planning will allow for a comprehensive understanding of the data gathered at these critical time intervals, thereby enhancing the overall credibility and reliability of the study findings.

## Timing of Outcome Assessments

The outcomes of ROSC, including both ROSC and sustained ROSC, and changes in mean arterial blood pressure will be evaluated during resuscitation efforts subsequent to the admission of participating patients to the ED. After achieving ROSC, survival to admission will be assessed based on the patient’s transition from ED to inpatient hospitalization. Survival to discharge and neurological prognosis will be evaluated at the time of discharge. Post-discharge follow-up evaluations for survival and neurological functionality will be conducted via telephone interviews at any given time in the week for the 1st, 3rd, and 6th months after ROSC. This comprehensive evaluation strategy aimed to accurately capture and analyze the complete progression and potential recovery trajectories of our study participants. To minimize loss to follow-up as much as possible, staff at each study site will make every reasonable effort to maintain contact with patients throughout the entire study period. This includes discussing their health status when assessing the Cerebral Performance Category (CPC) during phone calls.

## Statistical Principles

## Confidence Intervals and P-values

All statistical hypotheses in this study will be subjected to rigorous testing at a significance level of 0.05. This standard threshold for significance ensures the robustness of our findings and minimizes the likelihood of false–positive results. Furthermore, we reported our results with 95% confidence intervals for additional validity and precision. This commonly adopted approach underscores our confidence that the true population parameter lies within this defined range, thereby providing a reliable and comprehensive representation of our study findings.

## Adherence and Protocol Deviations

This study defined adherence to the intervention as the proportion of patients initially assigned to the REBOA CPR group who will have undergone aortic occlusion. This approach reflects real-world circumstances and captures the extent of exposure to the intervention, considering the time-sensitive nature of applying the REBOA upon hospital arrival. Adherence to the intervention will be quantitatively expressed in terms of percentages to depict the proportion of patients who actually received aortic occlusion in the REBOA CPR group.

Protocol deviations will be identified as instances in which patients in the REBOA group did not receive the aortic occlusion as planned. These deviations will be systematically assessed and summarized, focusing on the reasons why aortic occlusion was not achieved in the intervention group. A summary of protocol deviations will provide a detailed breakdown of the reasons for the deviation and their respective proportions within the intervention group.

## Analysis Populations

Two distinct analytical population sets will be used. The first is the intention-to-treat set, which examines patients based on their initial randomized group assignment, irrespective of the ultimately administered treatment. The second is the per-protocol set, which specifically analyzes patients who did (the REBOA group) and did not (the non-REBOA group) undergo the REBOA procedure.

The safety analysis for this study will encompass all patients who will have undergone an attempt at REBOA placement, which is characterized by any attempt to puncture the femoral vessels. This indicates regardless of whether the insertion of the REBOA catheter into the target vessel or accomplishment of aortic occlusion was successful. The purpose of this population was to critically evaluate the safety of implementing REBOA in a CPR scenario. This evaluation guarantees comprehensive accounting of the potential safety considerations associated with the intervention.

1. **Trial Population**

## Eligibility

- - 1. **Inclusion Criteria**

Patients aged 20–80 years with nontraumatic witnessed OHCA, arriving at the ED between 9AM to 5PM (in each country).

- - 1. **Exclusion Criteria**

The exclusion criteria are as follows: 1) patients aged below 20 years old or over 80 years old, 2) those with traumatic cardiac arrest; 3) those with unwitnessed cardiac arrest; 4) pregnant patients; 5) those who have already achieved ROSC upon arrival at the ED; 6) those with a precardiac arrest cerebral performance category (CPC) score of 3–4; 7) those showing evidence of cardiac arrest caused by bleeding (such as gastrointestinal bleeding); 8) those suspected of aortic disease, such as dissection, intramural hematoma, or aneurysm, by bedside ultrasound performed immediately after ED arrival or have a previous history of aortic disease; 9) those whose legal representatives request termination of resuscitation efforts before study enrollment; 10) those declared dead at scene before enrollment; and 11) those who meet the criteria for extracorporeal CPR (ECPR), and have been decided to receive ECPR. ECPR applies when all of the following criteria are met: precardiac arrest CPC score of 1–2; witnessed cardiac arrest with bystander CPR; age between 20–70; initial shockable rhythm; ECMO pump-on available within 60 minutes of the onset of cardiac arrest; and absence of end-stage diseases such as cancer, liver cirrhosis, or end-stage renal failure.

## Recruitment

Each hospital will assess all OHCA patients for eligibility and will continue until the target number of patients is reached. Enrollment will be continuously monitored to ensure that each hospital can enroll 50% of its total population. A minimum of 50 patients will be enrolled competitively to minimize racial differences. The Consolidated Standards of Reporting Trials (CONSORT) flowchart is as follows:


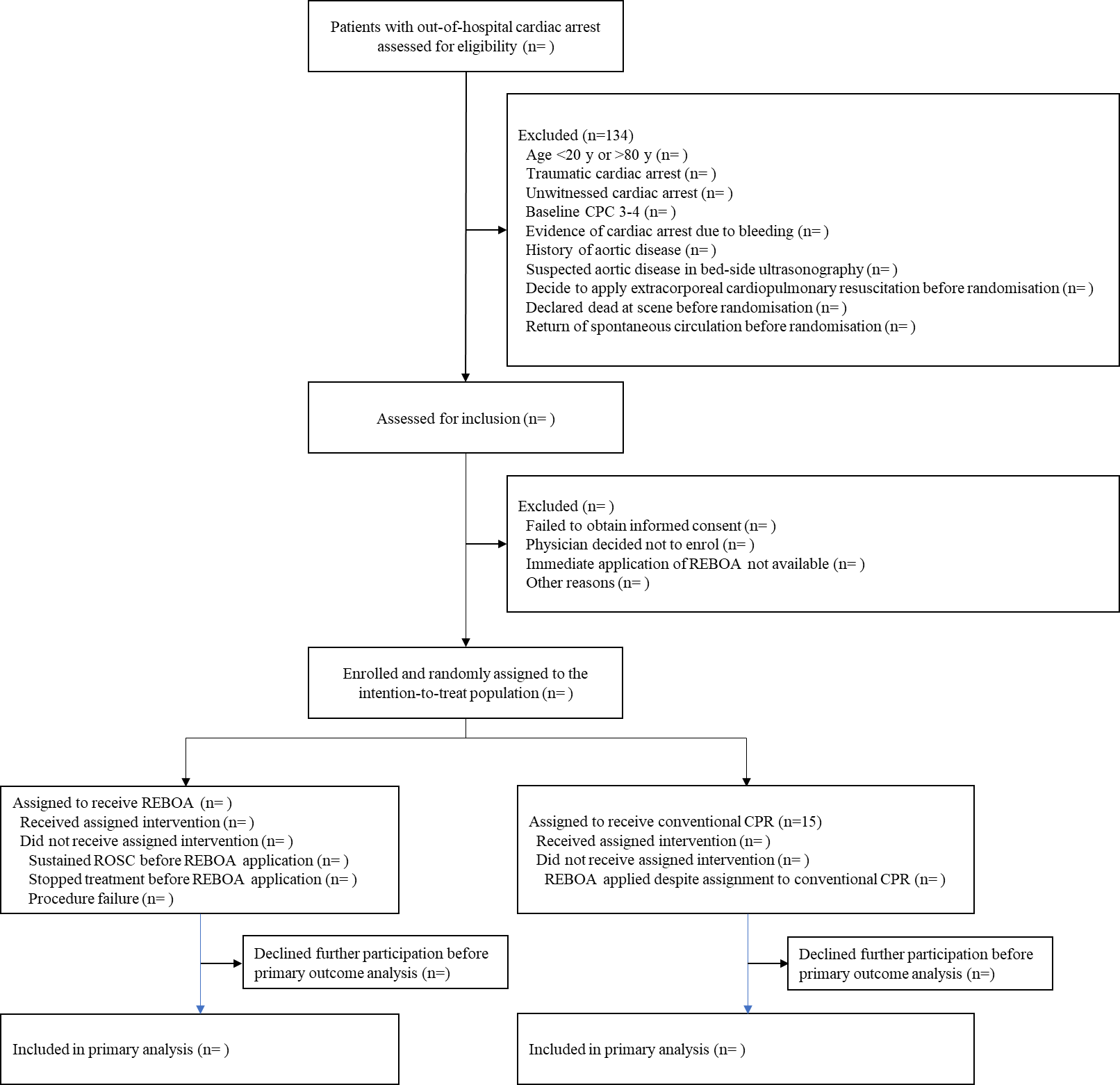


Figure 1. Consolidated Standards of Reporting Trials (CONSORT) flowchart

## Withdrawal or Loss Follow-up

The withdrawal level at every stage of the study, whether during the intervention or follow-up phase, will be quantified as a percentage. Any decision to withdraw from the study, made by either the trial participant or their legally authorized representative, will be meticulously recorded, encapsulating the reasons and precise timing of such actions. If a participant becomes inaccessible during the follow-up period, their data will be considered missing during the analysis. Ultimately, these exhaustive details regarding withdrawal will be incorporated into the CONSORT flow diagram to provide a transparent and integrated overview of participant retention throughout the course of the study.

## Baseline Patient Characteristics

Demographics (age, gender, and underlying disease), arrest data (timing and place of arrest, bystander CPR, initial rhythm, etc.), in-hospital data (arrival time, time of central/arterial line placement, etc.), outcome, and adverse events will be collected and are presented (Table 1).


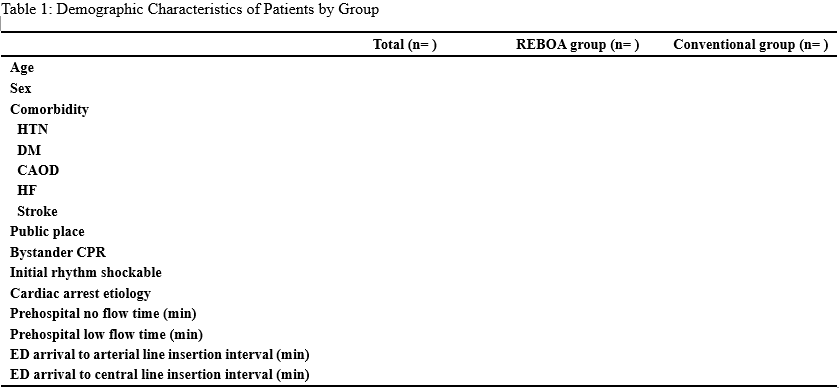


Data related to REBOA placement, including the time of placement, volume of balloon inflation, and depth of placement, will also be meticulously recorded and encapsulated within a table (Table 2) for a comprehensive review and reference.


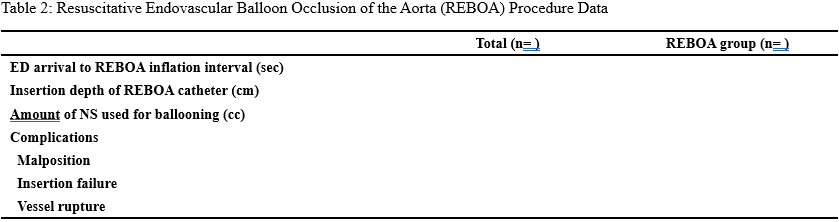


## Analysis

## Outcome Definitions

### **Primary Efficacy Endpoints**

### Any ROSC, defined as an occurrence in which a post-CPR patient establishes a palpable pulse that endures for a duration exceeding 1 min.

### **Secondary Efficacy Endpoints**

### Change in mean arterial blood pressure 1 min after balloon inflation.

### Sustained ROSC is defined as an occurrence in which a post-CPR patient establishes a palpable pulse that endures for a duration exceeding 20 min.

### Survival to hospital admission.

### Survival to hospital discharge.

### CPC score at the 1st, 3rd, and 6th month after ROSC.

### CPC scores were defined as follows:

- CPC 1: Good cerebral performance—The patient is conscious, alert, and able to work. There might be slight neurological or psychological deficit.
- CPC 2: Moderate cerebral disability—The patient is conscious and has sufficient cerebral function for independent activities of daily life. The patient could work in a sheltered environment.
- CPC 3: Severe cerebral disability—The patient is conscious but dependent on others for daily support because of impaired brain function.
- CPC 4: Coma or vegetative state—The patient is unconscious, with no meaningful interaction with the environment.
- CPC 5: Brain death—The patient had no measurable brain function.

## Analysis Methods

The Shapiro–Wilk test will be used to assess the normality of the data. Continuous data that follow a normal distribution will be presented as means and standard deviations and further analyzed using an independent t-test. In contrast, data not adhering to a normal distribution will be expressed as medians coupled with their interquartile ranges (IQRs) and evaluated using the Mann–Whitney U test. Numerical variables, such as the CPC score, will also be presented as medians with IQRs and analyzed using the Mann–Whitney U test. Categorical variables, including ROSC and survival, will be presented as frequencies (percentages) and analyzed using the chi-squared test or Fisher's exact test, as appropriate.

The difference in the two groups for variables showing statistically significant differences will be reported as either absolute difference or risk difference, depending on the type of variables. The primary outcome difference between the two groups will be analyzed using absolute difference and risk difference. Additionally, risk ratio will be presented as an additional measure, as appropriate.

Statistical significance was set at p value < 0.05. All the outcome analyses are presented in Table 3.


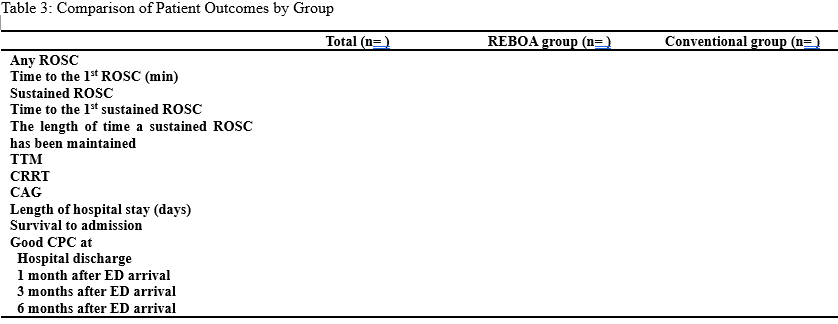


## Missing Data

We expect no missing data for the primary outcome of this study, given that it can be determined immediately when CPR is terminated, either due to ROSC or a determination of death in the EDs. Regarding the reporting of the clinical characteristics of patients and secondary outcomes, the primary approach to handling missing data is the direct deletion method. However, in cases where a significant amount of missing data exists for specific variables among the clinical characteristics, multiple imputation is considered an alternative approach. If multiple imputation is employed for any variable, it is explicitly stated in the reporting of results.

## Additional Analyses

The objective of the additional subgroup analysis is to investigate the factors potentially causing disparities in treatment outcomes, which may vary based on the distinct clinical characteristics of patients throughout the study. This detailed evaluation will be conducted for various potential determinants, including age, gender, whether CPR was administered by a bystander, initial rhythm (categorized as shockable or non-shockable), location of the arrest (public vs. non-public), cause of arrest (cardiogenic vs. non-cardiogenic), time elapsed from arrest to arrival at the ED, and the institution where the treatment was received (Seoul National University Bundang Hospital or Far Eastern Memorial Hospital).

## Harms

Adverse events will be monitored from randomization to hospital discharge. Any adverse events, including malposition, insertion failure, insertion site hematoma/infection, femoral artery injury, lower limb ischemia, aorta injury, and others, will be recorded in the case report form for each case. If any adverse events occur, patients will be treated immediately according to routine practice and will be followed up until complete resolution or termination of treatment. Such adverse events will be reported to the TSC and IRB.

## Statistical Software

Data will be analyzed using the R statistical software (version 4.2.3; R Core Team, R Foundation for Statistical Computing, Vienna, Austria).
