# Supplementary material for: Effect of resuscitative endovascular balloon occlusion of the aorta in nontraumatic out-of-hospital cardiac arrest: a multinational, multicenter, randomized, controlled trial
Source: Trials. 2024 Feb 13;25:118. doi: 10.1186/s13063-024-07928-x (PMC10863125; doi:10.1186/s13063-024-07928-x)
Supplement: Supplementary file 3 — Additional file 3. Informed consent (SNUBH). [file 13063_2024_7928_MOESM3_ESM.docx]

**Subject Information Sheet**

Subject Screening Number: ________________

**Title of the Study:** Effect Of Resuscitative Endovascular Balloon Occlusion of the Aorta in Non-Traumatic Out-of-Hospital Cardiac Arrest (REBOA); A Multinational, Multicenter Randomized Controlled Trial

**Principal Investigator:** Professor Dong Keon Lee, Department of Emergency Medicine, Bundang Seoul National University Hospital

We kindly request your participation in this study.

Before deciding to participate, it is important for you to fully understand why this study is being conducted and what your involvement will entail.

The following information is provided to explain the details of this study, your role in it, and the process of the research. Please take sufficient time to read this subject information sheet, and if desired, discuss it with your family or others. Additionally, if you have any questions, please ask the exam coordinator or other study personnel, and carefully consider your decision to participate in this study.

**1. This study is conducted for research purposes.**

This clinical trial is not conducted for therapeutic purposes. It is a research study aimed at investigating the impact of Resuscitative Endovascular Balloon Occlusion of the Aorta (REBOA) during cardiopulmonary resuscitation on the prognosis of out-of-hospital cardiac arrest patients, with the goal of improving the prognosis of cardiac arrest patients.

**2. Background and Purpose of the Clinical Trial**

For out-of-hospital cardiac arrest (OHCA), cardiopulmonary resuscitation (CPR) is performed. However, even with high-quality CPR, the rate of return of spontaneous circulation (ROSC) is extremely low. The primary goal of CPR is to maintain sufficient blood flow to the brain and coronary arteries, preventing ischemic damage and correcting the reversible causes of cardiac arrest. However, even with effective chest compressions, only 30% of the pre-arrest blood flow to vital organs is achieved. This study aims to increase the rate of ROSC by using REBOA during CPR, which involves occluding the descending aorta to increase blood flow to major organs during resuscitation.

**3. Information on Investigational Drugs/Medical Devices and Randomization Probability**

This study will be conducted using randomization. The assignment to either the CPR group or the REBOA group will be determined randomly based on a computer-generated randomization table, with an equal allocation ratio of 1:1. This study is a multinational study, with a total of 232 patients participating from Taiwan and Korea. If you agree to participate in the study, you will be randomly assigned to either the control or experimental group.

**4. Various Tests and Procedures that Subjects will Undergo in the Clinical Trial**

Both groups will receive CPR according to the 2020 American Heart Association guidelines for cardiopulmonary resuscitation. This standard CPR includes high-quality chest compressions at a rate of 100-120 per minute, prevention of hyperventilation, early defibrillation, and administration of medications such as epinephrine.


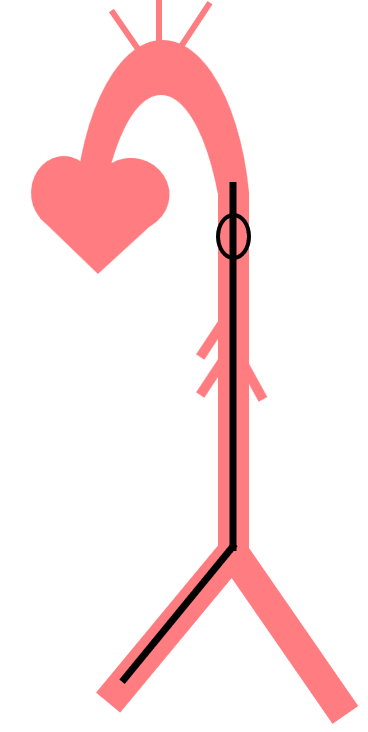
After the randomization process, if assigned to the control group, you will receive standard CPR as described above.

If assigned to the experimental group, in addition to the standard CPR according to the guidelines, femoral arterial access will be obtained, and a REBOA catheter will be inserted through the access. The catheter will be positioned within the aorta. The catheter will be inserted to an adequate length, and the balloon attached to the catheter will be positioned in the chest. Saline solution will be injected into the balloon to inflate it, obstructing the aorta, and preventing unnecessary blood flow to the lower body (Figure 1). Additionally, the catheter, arterial access, and other vital sign monitoring devices will be used to monitor hemodynamic parameters, including blood pressure, during CPR. If a return of spontaneous circulation is achieved, the REBOA will be removed after stabilization.

Figure 1. Schematic diagram of REBOA

The expected participation period for research subjects is one year, and they will be contacted by phone to measure neurological outcomes after treatment completion.

**5. Compliance requirements for research subjects**

There are no specific compliance requirements for research subjects. They may be contacted by phone to investigate neurological outcomes one year after resuscitation. Contact information for patients and caregivers will be collected for phone calls.

**6. The fact that it is an unproven clinical trial**

In cardiac arrest patients, conventional CPR is usually performed according to the American Heart Association CPR guidelines. This involves processes such as chest compressions, defibrillation, and administration of medications such as epinephrine. However, even when CPR is performed according to these guidelines, neurological outcomes are often poor due to ischemic brain injury caused by cardiac arrest.

This study is an unproven clinical trial aiming to increase the rate of spontaneous circulation recovery by using REBOA during CPR to block the descending aortic blood flow, which directs blood flow to major organs.

**7. Expected side effects, risks, or discomfort for subjects**

The following side effects may occur in this study:

Insertion-related vascular damage or aortic injury during the insertion process of the arterial sheath and REBOA may occur. To prevent this, the sheath and catheter will be inserted under ultrasound guidance using the Seldinger technique. REBOA will be inserted in a way that does not overlap with the chest compressions. Direct pressure will be applied to control bleeding if a hematoma occurs at the insertion site.

**8. Expected benefits for subjects**

REBOA is a procedure already being performed on trauma patients, and multiple animal experiments have shown improvements in hemodynamics in cardiac arrest patients. Considering previous research results, higher survival rates can be expected compared to performing conventional CPR alone. However, this study is an unproven clinical trial, so it is difficult to expect unconditional effectiveness.

**9. Alternative treatments (other available alternative treatments outside of the clinical trial)**

If not participating in this study, participants will receive standard CPR according to the 2020 American Heart Association guidelines. This includes chest compressions at a rate of 100-120 per minute, defibrillation, prevention of hyperventilation, and the use of medications such as epinephrine.

**10. Injury and compensation**

During the clinical trial period, the medical staff will make every effort to ensure the safety of the patients and take prompt and appropriate actions to minimize any adverse reactions. In the event of harm occurring during this clinical trial, the principal investigator of the study will assume legal responsibility and provide compensation according to the provisions regarding compensation for harm. In the case of adverse reactions, the best possible treatment will be provided.

**11. Financial compensation**

No financial compensation is provided for participants.

**12. Expected costs**

The consumables used in this study, such as the REBOA catheter, will be supported by the research budget. Other than the REBOA, procedures performed are the same as procedures administered during conventional CPR, so there will be no additional costs incurred by participating in the study.

**13.** **Voluntary participation**

Participating in the clinical trial is entirely up to you. If you do not agree to participate, it will not be a problem. Even after agreeing to participate in the trial, you can withdraw consent at any time without any disadvantages.

**14.** **Provision of personal information**

During and after the completion of the clinical trial, the monitor, inspector, and other authorized personnel may directly access research-related data, including medical records, within the scope prescribed by regulations, to verify the procedural integrity and reliability.

**15. Confidentiality:**

All records regarding your personal information obtained during this research will be kept confidential and will not be disclosed to anyone else. Even if the research results are published, your personal information will remain confidential.

**16. Continuous Provision of Research-related Information:**

During this research, if the investigator becomes aware of any new facts or information that might influence your decision to continue participation, they will inform you or your representative of such facts or information.

**17. Contact Information:**

You or your representative may contact the following individuals for telephone consultations at any time:

Person to consult regarding issues, concerns, or questions arising from the clinical trial:

Principal Investigator: Professor Dong Keon Lee ☎031-787-7579

Co-Investigator: Hee Eun Kim ☎031-787-7579

Contact information for discussing issues, concerns, or questions related to the rights of research participants:

IRB (Institutional Review Board) Support Office ☎031-787-8801~8806

Clinical Research Ethics Center ☎031-787-88118813

**18. Termination of the Study:**

If any clinically significant adverse events occur during this clinical trial that are believed to be not beneficial to the patient, the patient may be withdrawn from the study.

**19. Expected Duration of the Participant's Clinical Trial:**

The anticipated duration of the participant's involvement in the clinical trial is one year, and they will be contacted by phone to assess their neurological prognosis after treatment completion.

**20. Number of Participants in the Clinical Trial:**

This is a multi-country, multi-center study. A total of 232 participants are expected to participate. Our institution plans to enroll 116 participants.

**21. Miscellaneous:**

If you decide to participate in the clinical trial, please fill in the provided consent form.

**Consent Form for Research Participants**

Title of the Study: Comparison of REBOA Use and Conventional CPR in Out-of-Hospital Cardiac Arrest Patients

1. I have received a detailed explanation from the responsible physician regarding all information related to this research and have understood it sufficiently.
2. I have also read the participant information sheet and understood its contents adequately, and I am aware that this research is being conducted for research purposes.
3. My decision to participate in the research is voluntary, and I understand that I can refuse to continue participating or withdraw freely during the research period due to personal reasons or other factors, without receiving any medical or other disadvantages as a result.
4. I am aware that if I experience harm due to adverse reactions caused by investigational medicinal products, the "compensation responsible party" will bear responsibility for compensation according to the provisions related to compensation.
5. I understand that I may receive telephone contact for the purpose of investigating neurological prognosis up to one year after resuscitation, and I consent to the research accessing my contact information and personal details for research purposes only.
6. I understand that I can contact the investigator at any time if I have any questions related to the research, and I agree to allow direct access to my medical records for research purposes only.

Accordingly, I voluntarily agree to participate in this research according to my free will.

Please sign and date below in your own handwriting.

|  | **Name** | **Signature** | **Signed Date** |
| --- | --- | --- | --- |
| **Participant** |  |  | YYYY / MM / DD |
| **Legal Representative** |  |  | YYYY / MM / DD |
|  | Relationship with the Participant: ________________________  Reasons for Proxy Signature ____________________________________________ | | |
| **Principal investigator** |  |  | YYYY / MM / DD |

If applicable,

|  | **Name** | **Signature** | **Signed Date** |
| --- | --- | --- | --- |
| **Observer** |  |  | YYYY / MM / DD |
