# Supplementary material for: Effect of resuscitative endovascular balloon occlusion of the aorta in nontraumatic out-of-hospital cardiac arrest: a multinational, multicenter, randomized, controlled trial
Source: Trials. 2024 Feb 13;25:118. doi: 10.1186/s13063-024-07928-x (PMC10863125; doi:10.1186/s13063-024-07928-x)
Supplement: Supplementary file 4 — Additional file 4. Informed consent (FEMH). [file 13063_2024_7928_MOESM4_ESM.pdf]

Far Eastern Memorial Hospital

Informed Consent Form for Clinical Trials of Medical Devices

Project Title: Resuscitative Endovascular Balloon occlusion of the Aorta in Patients with Non-Traumatic Out-of-Hospital Cardiac Arrest - An International Multi-center Randomized Clinical Trial

Clinical Trial Institution: Far Eastern Memorial Hospital

Commissioner: Jen-Tang Sun

Project host: Jen-Tang Sun

Position: Director of Surgical and Traumatic Emergency / Tel: 02-77281843

Co-host: Chun-Yen Huang / Position:Attending physician / Tel: 02-77281843

Co-host: Chih-Jung Chang / Position: Attending physician / Tel: 02-77281843

Co-host: Sheng-En Chu / Position: Attending physician / Tel: 02-77281843

Co-host: Yu-Chen Chiu / Position: Attending physician / Tel: 02-77281843

Co-host: Fu-Chieh Hsieh / Position: Attending physician / Tel: 02-77281843

※**24-hour emergency contact:** Jen-Tang Sun **Tel: 0919-388100**

Name:

Gender:

Medical record number:

You are cordially invited to participate in this clinical trial. This document provides you with pertinent information concerning the study. The lead investigator or an authorized representative will thoroughly explain the trial's objectives and address any inquiries you might have. Please ensure that all your questions are answered to your satisfaction before affixing your signature to this consent form. We understand the significance of such a decision, so we encourage you to reflect and deliberate before committing. Your signed consent is a prerequisite for participation in this trial. Should you choose to partake, this document will stand as an official record of your agreement. It's essential to note that even

after giving your consent, you retain the right to withdraw from the study at any point, with no need to furnish a reason.

1. Status of the Medical Device Used in the Trial:

Information on the Medical Device:

The Resuscitative Endovascular Balloon Occlusion of the Aorta (commonly referred to as REBOA) is currently considered suitable for severe hemorrhaging below the diaphragm or even impending traumatic cardiac arrest (ITCA). It offers a minimally invasive, quick, and effective alternative surgery for bleeding control. This technique can halt hemorrhagic shock and prevent the exacerbation of metabolic issues, simultaneously providing patients with vital time, avoiding rushed, potentially unprepared invasive procedures like thoracotomy, laparotomy, or pelvic cavity tamponade in the emergency department. The method involves introducing a catheter into the aorta through a femoral approach and inflating a balloon near the bleeding site to temporarily halt blood flow, thereby increasing afterload to maintain cerebral and cardiac circulation. The aorta can be segmented into three zones: Zone I spans from the left subclavian artery to the celiac trunk, controlling major hemorrhage below the diaphragm; Zone III ranges from the renal arteries to the aortic bifurcation, managing major bleeding at the pelvis or lower limbs; Zone II, which is the region between Zones I and III, presently has limited applicability. Employing this technique can result in ischemia in organs distal to the balloon, potentially leading to complications such as visceral, spinal cord, and lower limb ischemia or reperfusion syndrome. Numerous studies on the use of REBOA in severe trauma patients have shown a 30-day survival rate of 59%. Traumatic cardiac arrest patients using REBOA to restore spontaneous circulation stand at 58.8%, with 40-41% reaching the operating room alive, and a 30-day survival rate between 5-10%. Beyond traumatic shock, REBOA is gradually being used for non-traumatic hemorrhage cases. According to Matsumura's study, common reasons for using REBOA include postoperative hemorrhage, ruptured aneurysm, gastrointestinal bleeding, and major postpartum hemorrhage. Although the mortality rate for these patients matches that of traumatic shock patients, the proportion of deaths due to bleeding is noticeably reduced. Far Eastern Memorial Hospital recorded two cases of aneurysm hemorrhage and two postpartum major hemorrhage cases. Among these, three had cardiac arrest, all of whom were resuscitated. One of the postpartum hemorrhage patients was successfully discharged with full neurological functionality. The team has published a case of ruptured aortic aneurysm treated with

REBOA in the Annals of Emergency Medicine.

Market Status of the Medical Device:

The device has received approval for marketing by the Taiwan Ministry of Health and Welfare and meets the indicated use of temporary vascular occlusion or dilation of artificial vessels (Health Ministry Medical Equipment Import No. 032086).

2. Objective of the Study:

Driven by our expertise in resuscitation for patients with Out-of-Hospital Cardiac Arrest (OHCA), and supported by positive evidence from past literature on the use of REBOA in OHCA patients, along with our team's extensive experience with REBOA, we aim to apply REBOA in the emergency management of OHCA patients. In our study, we explore the potential of using REBOA to assist with Advanced Cardiac Life Support (ACLS) in the hospital setting, an area less emphasized in many Western research studies that often prioritize pre-hospital care. We are proposing a three-year continuous project that will be a randomized controlled study across two international medical centers. Our research hypothesis posits that adult witnessed non-traumatic cardiac arrest patients, when aided with REBOA technique in conjunction with ACLS, as opposed to using ACLS alone, will exhibit better rates of return of spontaneous circulation, improved end-tidal carbon dioxide levels, higher non-invasive cerebral oxygenation levels, and patients will also display superior arterial perfusion, overall survival rates, and neurological prognosis. For this study within our country, we anticipate to include a control group of 58 patients and a REBOA group of 58 patients, totaling 116 participants.

3. Inclusion and Exclusion Criteria:

Inclusion Criteria:

(1) Age between 20 and 80 years.

(2) Non-traumatic and non-hemorrhagic cause of the condition.

(3) Patients who experience witnessed cardiac and respiratory arrest between 9 am and 5 pm from Monday to Friday and are brought to the emergency department.

Exclusion Criteria:

(1)Patients or their representatives who have expressed refusal for resuscitation, either through a signed "Do Not Resuscitate" (DNR) order or an Advanced Care Planning (ACP) that explicitly states an opposition to emergency resuscitation efforts.

(2)Patients who have already achieved ROSC before arrival to the emergency department.

(3)Initiation of extracorporeal cardiopulmonary resuscitation.

(4)Clinical identification of aortic conditions, such as aortic dissections or aneurysms, as evidenced by ultrasound results or a documented history.

(5)Patients who were already severely neurologically impaired or in a vegetative state before the event.

(6)Pregnant women

4. Methodology & Procedures

REBOA Group Procedure:

The on-duty team treats the patient, including ACLS. Study-specific procedures are performed by research team physicians without affecting the original treatment.

(1) The resuscitation process is performed and recorded by the on-duty emergency medical team.

(2) Resuscitation following ACLS guidelines, including LUCAS treatment.

(3) Secure airway establishment (endotracheal intubation) and monitor end-tidal CO2 (EtCO2).

(4) Research team personnel conduct ultrasonography to rule out contraindications.

(5) Research team personnel insert femoral artery and central venous catheters to monitor arterial blood pressure and right atrial pressure.

(6) Non-invasive cerebral oxygenation monitoring is set up by the research team.

(7) Patient grouping (REBOA or Control) is determined using the Redcap system.

(8) If assigned to the REBOA group, the research team places the REBOA between the left subclavian artery and the abdominal aorta main (Zone I), confirmed by transesophageal echocardiography, without interrupting CPR, and arterial pressure is

monitored.

(9) Inflate the REBOA balloon with 20cc saline or until resistance is felt.

(10) Continuous monitoring until the patient achieves Return of Spontaneous Circulation (ROSC) or is declared deceased.

(11) Post-ROSC, the REBOA balloon is gradually deflated and removed. Post-resuscitation care is provided, and a cardiothoracic surgeon is consulted for arterial catheter removal evaluation.

(12) Record patient details, physiological parameters, resuscitation measures, REBOA-related info (attempts, insertion time, complications, CCF, and other ACLS quality indicators).

(13) If the patient achieves sustained ROSC (more than 20 minutes), they sign a consent form. Tracking continues for 30 days post-enrollment or until death. Patient survival status and neurological outcomes are recorded.

Control Group Procedure:

The on-duty team treats the patient, including ACLS. Study-specific procedures are performed by the research team physicians without affecting the original treatment.

(1) to (6) steps are similar to the REBOA group (from emergency procedure to cerebral oxygenation monitoring).

(7) Group assignment is made using the Redcap system.

(8) If assigned to the Control group, REBOA is not inserted.

(9) Monitoring continues until the patient achieves ROSC or is declared deceased.

(10) to (11) steps are consistent with the REBOA group, focusing on ROSC achievement, post-resuscitation care, and consultation with a cardiothoracic surgeon.

(12) to (13) steps. Similarly, record patient details, parameters, resuscitation efforts, and monitor patient's health and neurological outcomes for 30 days post-enrollment or until death.

5. Potential Risks, Occurrence Rates, and Management:

Risks Associated with the Medical Device Used in the Study:

The complications may arise from the REBOA itself or during its placement process. These include but are not limited to: infections at the injury site or the onset of sepsis, allergic reactions, localized bleeding, vasospasms, endothelial injury, air embolism, pain and tenderness at the site of insertion, peripheral vascular ischemia, endocarditis, thrombocytopenia, myocardial infarction, blood clots leading to vascular obstruction, cerebral embolism, arteriovenous fistula, kidney-related complications, vascular complications (dissection, rupture, bleeding, or perforation), respiratory distress, or death.

Risks Related to the Trial Procedure:

The use of REBOA on eligible participants has been optimized to minimize risks, with every case being reviewed by a cardiothoracic surgeon. Despite this, there remains a possibility of participant death even after the standard resuscitation procedure. The primary responsibility of the cardiothoracic surgeon is to assist with the removal of the arterial catheter sheath and to be on standby in cases of difficulty with REBOA removal.

6. Alternative Therapies and Explanation:

Standard Advanced Cardiac Life Support (ACLS) without the placement of REBOA.

7. Anticipated Benefits of the Trial:

The trial aims to increase coronary and cerebral perfusion in participants. In cases where aggressive treatments (such as ECMO) are not applicable, it hopes to enhance the chances of participants achieving return of spontaneous breathing and circulation, as well as improving neurological outcomes.

8. The Contraindications, Limitations, and Requirements for Participants During the Trial:

None

9. Confidentiality of Participants' Personal Information:

The system data is maintained by a dedicated research assistant, ensuring confidentiality

of all relevant information. The project leader takes meticulous steps to protect the collected documents and complies with the "Personal Data Protection Act" and other related regulations. Initially, the data will be labeled with the participant's name and medical record number. The participants will then be coded. Once the subsequent medical record data collection is completed (within 30 days), the names of the participants will be removed, leaving only the code. All case data will be archived on the research computers of both the lead and co-investigators.

After undergoing research coding management, the analyzed data completely omits personal identifiers such as national identification numbers, home phone numbers, addresses, etc., making it impossible to trace the patient's identity, ensuring the privacy of the subjects. Collected hard copies and multimedia data will be stored on the assistant's computer. The lead investigator will personally analyze them, or research members will analyze them on the computer under the supervision of the lead investigator.

10. Withdrawal and Termination of the Trial:

You are free to decide whether to participate in this trial. You can also withdraw your consent and leave the trial at any time, without providing any reason. This decision will not result in any inconvenience and will not affect the future medical care provided to you by your physician. The trial's lead investigator may also choose to terminate the trial if deemed necessary.

11. Medical Care, Compensation, and Insurance:

(1) If you participate in this clinical trial and sustain damage due to an adverse reaction, our hospital will bear the responsibility for compensation. However, anticipated adverse reactions, side effects, or risks noted in this consent form will not be compensated.

(2) If you participate in this clinical trial and experience an adverse reaction or damage, our hospital and the lead investigator are committed to providing professional medical care and consultation. You will not be responsible for the necessary medical expenses associated with treating the adverse reaction or damage.

(3) Aside from the aforementioned compensation and medical care, this trial does not offer any other form of compensation. If you are unwilling to accept these risks,

please refrain from participating in the trial.

(4) Signing this consent form will not deprive you of any legal rights.

(5) This trial does not have liability insurance.

12. Preservation, Use, and Reuse of Participant's Specimens (and their derivatives) and Personal Information:

The collected data will be coded and stored on the computers of the lead and co-investigators, managed by the aforementioned personnel. Only the participant's code will be displayed on the collected data. Additionally, all data gathered during this study, including paper records and video recordings, will be destroyed within 3 years after the conclusion of the study.

13. Rights of the Participant:

(1) There will be no charges related to this study imposed on you, and this trial is not covered under the National Health Insurance scope.

(2) Any significant findings related to your health or illness that may affect your willingness to continue participating in the clinical trial will be promptly communicated to you.

(3) If you have questions about the nature of the trial or concerns about your rights as a participant, or if you suspect any harm as a result of participating in the trial, you can contact the Institutional Review Board (IRB) of our hospital for consultation at 02-89667000, extension 2152. Alternatively, you can contact the Participant Protection Center at 02-89667000, extension 2546.

(4) To proceed with the trial, you must be under the care of the physicians involved in this research. After being admitted to the Intensive Care Unit (ICU), you will be cared for by the ICU physicians. If you have any concerns or issues now or during the trial, please feel free to contact Dr. Sun Ren-Tang from the Emergency Medicine Department at Far Eastern Memorial Hospital, **available 24/7 at 0919-388100**.

(5) Two copies of this consent form have been provided, one of which has been handed to you. The physician has explained the nature and objectives of this trial in full and

has answered all your questions concerning the medical equipment and trial procedures.

14. Signature:

The principal investigator or their authorized personnel has explained in detail the nature, purpose, potential risks, and benefits of the trial method mentioned in this clinical trial.

□Principal Investigator/□Co-Investigator/□Authorized Research Personnel

Signature: ______________

Date: ____Year____Month____Day

I have fully understood the aforementioned trial method, its potential risks and benefits. Any questions about this trial have been thoroughly explained by the research team. I voluntarily agree to become a participant in this clinical trial.

Participant's Signature: ______________

Date: ____Year____Month____Day

Legal Representative/Person with Consent Authority Signature: ______________

Date: ____Year____Month____Day

Relationship to Participant: □Spouse, □Adult Child, □Parent, □Sibling, □Grandparent

* *If the participant is a teenager aged 7 to 20 with limited legal capacity, both the participant and the legal representative should sign.*
* *The legal representative should also present relevant identification documents for the principal investigator to verify their identity.*
* *Even if the participant is not legally incapacitated or has restricted capacity, should they exhibit signs of confusion, mental, or cognitive impairments that prevent effective communication and sound judgment, a person authorized to provide consent can act on their behalf. When proceeding to sign the consent form, the authorized person must*

present relevant identification documents to allow the principal investigator to validate their identity.

* *For written consents provided by the aforementioned relatives or authorized persons, a single person's signature suffices. In the event of conflicting opinions among these individuals, priority is determined by the predefined sequence, as indicated in the aforementioned order. Within the same category of relationship, closer kinship takes precedence. If kinship level is identical, cohabitating family members are given priority. In the absence of cohabitating relatives, the elder individual's decision takes precedence.*

Witness 1. Signature: _____________

Date: ____Year____Month____Day

ID Number:

Address:

Contact Number:

Witness 2. Signature: _____________

Date: ____Year____Month____Day

ID Number:

Address:

Contact Number:

* *If the participant, legal representative, or person with the right to consent is illiterate, a witness should be present throughout all discussions related to the consent form. After ensuring that the consent of the participant, legal representative, or person with the right to consent is given voluntarily, they should sign the consent form and record the date. Research-related personnel cannot act as witnesses.*
